# Terpene metabolic engineering *via* nuclear or chloroplast genomes profoundly and globally impacts off‐target pathways through metabolite signalling

**DOI:** 10.1111/pbi.12548

**Published:** 2016-03-08

**Authors:** Elise K. Pasoreck, Jin Su, Ian M. Silverman, Sager J. Gosai, Brian D. Gregory, Joshua S. Yuan, Henry Daniell

**Affiliations:** ^1^ Department of Biochemistry School of Dental Medicine University of Pennsylvania Philadelphia PA USA; ^2^ Department of Biology University of Pennsylvania Philadelphia PA USA; ^3^ Department of Plant Pathology and Microbiology Texas A&M University College Station TX USA

**Keywords:** Secondary metabolism, retrograde/anterograde signalling, transgenic/transplastomic plants, squalene

## Abstract

The impact of metabolic engineering on nontarget pathways and outcomes of metabolic engineering from different genomes are poorly understood questions. Therefore, squalene biosynthesis genes *FARNESYL DIPHOSPHATE SYNTHASE* (*FPS*) and *SQUALENE SYNTHASE* (*SQS*) were engineered *via* the *Nicotiana tabacum* chloroplast (C), nuclear (N) or both (CN) genomes to promote squalene biosynthesis. SQS levels were ~4300‐fold higher in C and CN lines than in N, but all accumulated ~150‐fold higher squalene due to substrate or storage limitations. Abnormal leaf and flower phenotypes, including lower pollen production and reduced fertility, were observed regardless of the compartment or level of transgene expression. Substantial changes in metabolomes of all lines were observed: levels of 65–120 unrelated metabolites, including the toxic alkaloid nicotine, changed by as much as 32‐fold. Profound effects of transgenesis on nontarget gene expression included changes in the abundance of 19 076 transcripts by up to 2000‐fold in CN; 7784 transcripts by up to 1400‐fold in N; and 5224 transcripts by as much as 2200‐fold in C. Transporter‐related transcripts were induced, and cell cycle‐associated transcripts were disproportionally repressed in all three lines. Transcriptome changes were validated by qRT‐PCR. The mechanism underlying these large changes likely involves metabolite‐mediated anterograde and/or retrograde signalling irrespective of the level of transgene expression or end product, due to imbalance of metabolic pools, offering new insight into both anticipated and unanticipated consequences of metabolic engineering.

## Introduction

Many metabolic engineering strategies rely on transformation of the nuclear genome. For example, avian *FARNESYL DIPHOSPHATE SYNTHASE* (*FPS*) and yeast *SQUALENE SYNTHASE* (*SQS*) have been expressed in *Nicotiana tabacum* to promote squalene accumulation (Wu *et al*., 2012). New metabolic pathways have also been engineered, for example by introducing the pathway for the cyanogenic glucoside dhurrin into *Arabidopsis* (Tattersall *et al*., 2001). Several other studies have found that directing key enzymes to chloroplasts can substantially increase the content of metabolites such as β‐carotene—used to produce ‘golden rice’ (Ye *et al*., 2000)—and terpenes, including patchoulol (Wu *et al*., 2006) and squalene (Wu *et al*., 2012).

However, nuclear transformation is associated with several disadvantages. Positional effects can reduce or silence transgene expression, making it difficult to establish a stable population that consistently accumulates high levels of the desired product (Jin and Daniell, 2015; Verma and Daniell, 2007). Additionally, lack of transgene containment raises regulatory issues and makes commercialization more challenging (Chan and Daniell, 2015). Some of these problems can be overcome by expressing transgenes *via* the chloroplast genome. Chloroplast transformation, which occurs through homologous recombination, contains transgenes (Verma *et al*., 2008), and its high ploidy—up to 10 000 copies per cell—permits transgene products to reach exceptionally high levels (Ruhlman *et al*., 2010). For example, tobacco chloroplasts expressing a multigene operon encoding Bt toxin allowed the protein to accumulate up to ~50% of total leaf protein and to form cuboidal crystals within chloroplasts (De Cosa *et al*., 2001); chloroplast‐expressed β‐glucosidase accumulated to 160‐fold higher levels than in untransformed plants (Jin *et al*., 2011); and proinsulin could make up nearly 70% of total transplastomic leaf protein (Ruhlman *et al*., 2010).

Chloroplast genome transformation has also been successfully used in metabolic engineering (Jin and Daniell, 2015): chloroplasts expressing chorismate pyruvate‐lyase accumulated the biopolymer *p*‐hydroxybenzoic acid to ~26.5% dry weight—the highest level reported for any bioproduct—without compromising plant health (Viitanen *et al*., 2004). In later studies, the chloroplast genome was engineered to express all six genes of the cytoplasmic mevalonate pathway for terpenoid synthesis (Kumar *et al*., 2012) or thirteen artemisinin pathway biosynthesis genes (Saxena *et al*., 2014), although in the latter case, expression was inadequate to meet target levels. However, chloroplast genome transformation requires further optimization, and a comparison of metabolic engineering outcomes *via* engineering different cellular compartments has not been performed.

The chloroplast genome is highly reduced, with many genes lost or transferred to the nucleus (Jensen and Leister, 2014). Consequently, chloroplast function requires the import of thousands of nuclear‐encoded proteins, many of which work in concert with plastid‐encoded gene products and require proper stoichiometry (Jin and Daniell, 2015). Therefore, the expression of nuclear‐ and plastid‐encoded genes must be coordinately regulated, and this occurs *via* anterograde signalling from the nucleus to plastids and retrograde signalling from plastids to the nucleus. Whereas anterograde signalling is well understood, retrograde signalling is still enigmatic. Chloroplasts may regulate nuclear gene expression *via* proteins (Jin and Daniell, 2014; Singh *et al*., 2008), redox state (Nott *et al*., 2006) or metabolites (Chi *et al*., 2015; Estavillo *et al*., 2011; Woodson *et al*., 2011; Xiao *et al*., 2012), but these signals act only under specific circumstances, and mechanisms by which they are conveyed remain elusive. However, high accumulation of proteins expressed *via* the chloroplast genome and compartmentalization within chloroplasts make chloroplast genetic engineering an excellent system to study retrograde signalling.

Much work on metabolic engineering and synthetic biology has focused on engineering pathways to generate high‐value metabolites, but the global impact of such engineering has not yet been explored despite the potential for unintended consequences (Bobik and Burch‐Smith, 2015). The availability of modern tools to study the metabolome and transcriptome facilitates global evaluation of the effect of these introduced pathways on native genes through metabolite‐mediated anterograde or retrograde signalling. Here, we used chloroplast genetic engineering and an existing nuclear transgenic line to uncover potential unintended consequences of expressing metabolic genes from different compartments. We focused on squalene because of its importance in steroid biosynthesis, its industrial applications in cosmetics and nutraceuticals (Kim and Karadeniz, 2012) and its use as a vaccine adjuvant (O'Hagan *et al*., 2011), as well as the availability of nuclear transgenic lines expressing squalene biosynthetic enzymes for comparative investigations (Wu *et al*., 2012). We found profound but similar off‐target effects on both the metabolome and transcriptome regardless of the compartment from which transgenes were expressed. By focusing on global effects of intercompartmental signalling rather than attempting to identify yet another signalling molecule, we provide a framework for future studies on large‐scale effects of metabolite‐mediated intercompartmental signalling.

## Results

### Characterization of transplastomic lines expressing *FPS* and *SQS*

**Figure 1:**
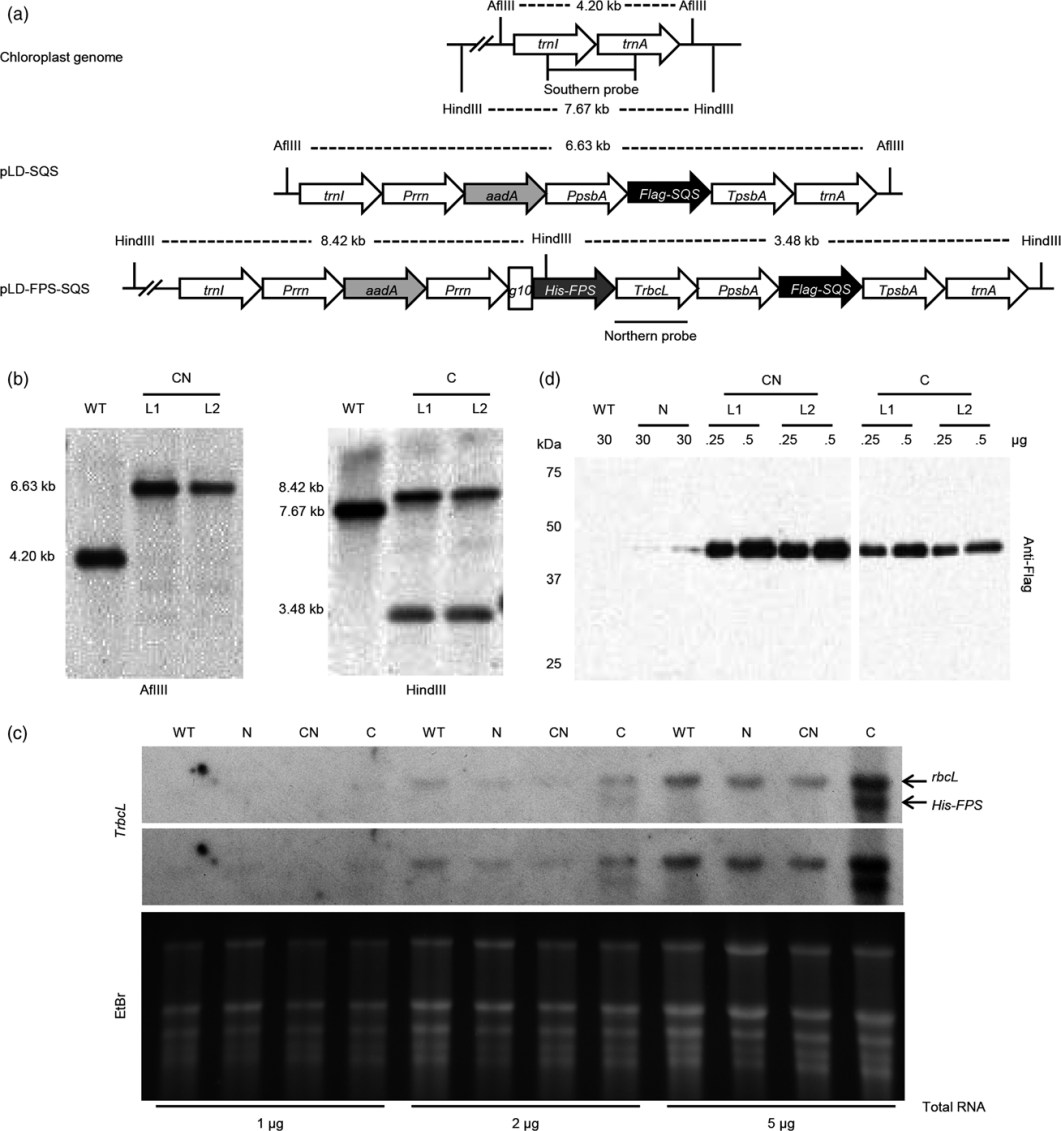
Chloroplast transformation vectors and characterization of transplastomic lines. (a) Schematics of the tobacco chloroplast genome, transformation vectors and probe hybridization sites with predicted sizes of hybridizing fragments in Southern blots. Top, untransformed; centre, pLD‐SQS; bottom, pLD‐FPS‐SQS. (b) Genomic DNA (0.5 μg) from independent transplastomic lines was digested with *Afl*III (CN lines, left) or *Hin*dIII (C lines, right) and probed with a DIG‐labelled probe specific for the *trnI/trnA* flanking region. (c) Total RNA (1–5 μg) was hybridized with a DIG‐labelled *TrbcL* probe. Northern blots were exposed to films for 15 min. (top) or 1 h. (middle), and ethidium bromide‐stained gel (bottom) is a loading control. (d) Anti‐Flag western blot of indicated amounts of total protein. FPS, *FARNESYL DIPHOSPHATE SYNTHASE;* SQS*, SQUALENE SYNTHASE*.

To engineer squalene biosynthesis through different organellar genomes, we generated two tobacco chloroplast expression vectors, one encoding Flag‐tagged SQS (pLD‐SQS) and one encoding both Flag‐tagged SQS and His‐tagged FPS (pLD‐FPS‐SQS) (Figure 1a). Encoded amino acid sequences were identical to those reported previously (Wu *et al*., 2012), but corresponding DNA sequences were codon‐optimized for enhanced chloroplast expression (Daniell *et al*., 2009). In pLD‐SQS, *Flag‐SQS* was regulated by the tobacco *psbA* promoter, 5′‐UTR and 3′‐UTR, and isoleucine tRNA (*trnI*) and alanine tRNA (*trnA*) flanking sequences were included for integration into the chloroplast genome *via* homologous recombination (Verma and Daniell, 2007). In pLD‐FPS‐SQS, expression of *His‐FPS* was controlled by the plastid rRNA operon promoter (*Prrn*), the 5′ translation control element of bacteriophage T7 gene *10* and the tobacco *rbcL* 3′‐UTR (Dhingra *et al*., 2004).

Two *N. tabacum* lines were used: the wild‐type (WT) 1068 introduction, which has abundant glandular trichomes that may be squalene sinks (Wu *et al*., 2012), and a transgenic line in the WT background expressing chloroplast‐targeted SQS and FPS *via* the nuclear genome (Wu *et al*., 2012), hereafter referred to as ‘N’. Leaves of N were bombarded with pLD‐SQS to generate transplastomic lines expressing *SQS* from the chloroplast genome; the resulting line is referred to as ‘CN’. After selection and regeneration on spectinomycin‐containing media, several independent CN lines were regenerated from ten bombardments. PCR analysis with the 3P/3M primer set (Verma *et al*., 2008) indicated that the *Flag‐SQS* cassette had been stably integrated into the chloroplast genome *via* homologous recombination (Figure S1). After two additional rounds of selection on spectinomycin‐containing media, CN lines were confirmed by Southern blot. As shown in Figure 1a,b, several independent CN lines showed a 6.63‐kb band but not the 4.43‐kb WT band, confirming that homoplasmic plants had been generated. We also bombarded N with pLD‐FPS‐SQS, but no shoots survived. When pLD‐FPS‐SQS was used to transform WT tobacco, several independent transplastomic lines, referred to as ‘C’, were obtained, as confirmed by PCR (Figure S1) and Southern blot (Figure 1b, left). Two bands with correct sizes of 8.42 and 3.48 kb (due to the presence of a *Hin*dIII site in the *His‐FPS* cassette), but not the 7.67‐kb WT band, were observed in *Hin*dIII‐digested DNA from C lines, confirming that homoplasmic C lines had been generated (Figure 1b, right). Because the engineered His tag was not detectable in western blots, we confirmed expression of *His‐FPS via* the chloroplast genome using northern blot for the *rbcL* 3′‐UTR (Figure 1a). In addition to the endogenous *rbcL* signal in each line, the C line showed an additional band corresponding to the expressed *His‐FPS* transgene (Figure 1c). Notably, the *His‐FPS* transcript was as abundant as that of *rbcL*, which is the most abundant protein on earth (Dhingra *et al*., 2004).

Expression of Flag‐SQS protein was detected by western blot using a monoclonal anti‐Flag antibody. A Flag signal could be detected in both transgenic and transplastomic lines. However, detection of the signal in N required loading as much as 120‐fold more protein (Figure 1d). To quantify this difference, we performed densitometric analysis. After accounting for differences in the amount of loaded protein and normalizing to N, we found that CN expressed 2813–4372 times more Flag‐SQS than N and that C expressed 1399–2309 times more than N.

### The impact of *FPS* and *SQS* expression on leaf and flower development

**Figure 2:**
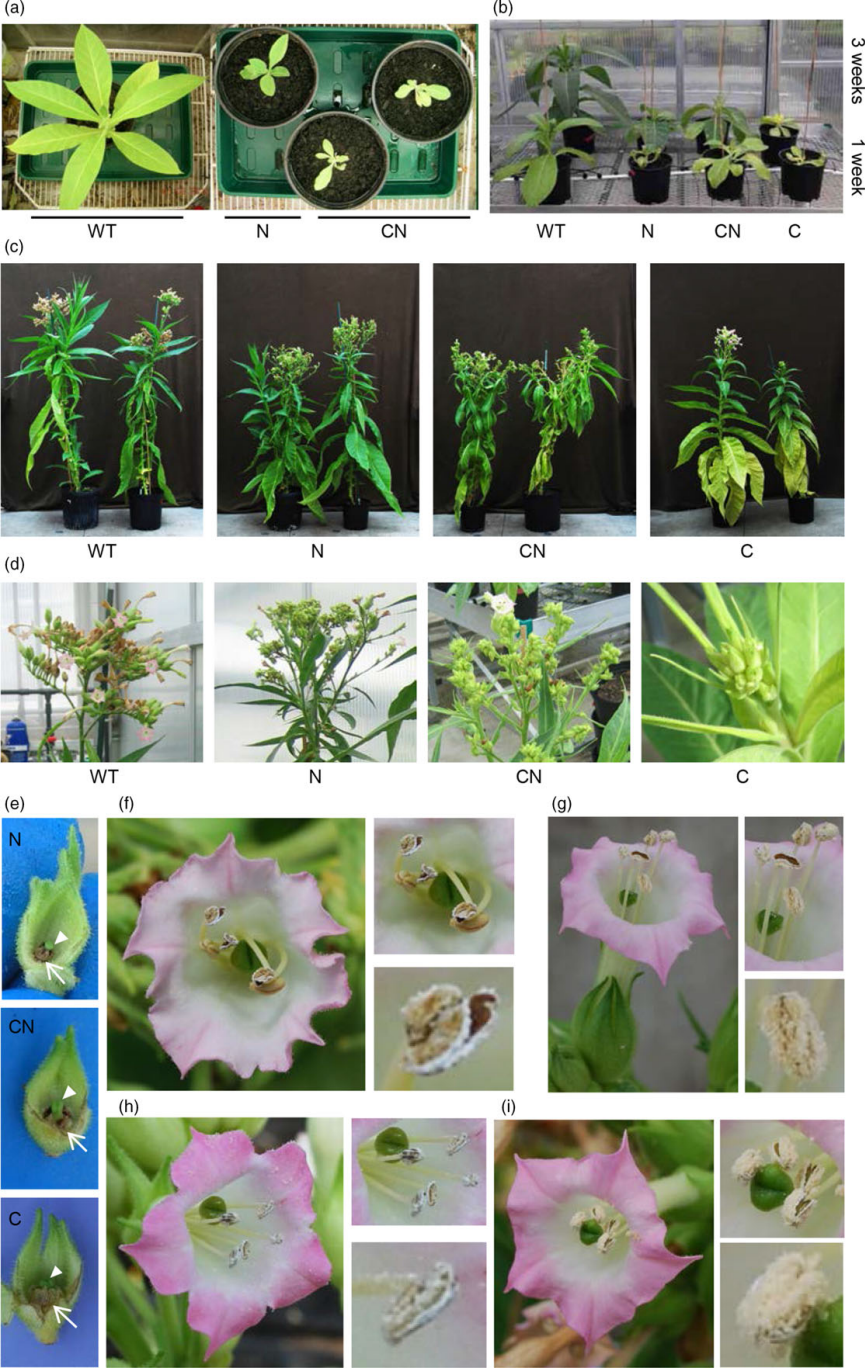
Leaf and flower phenotypes of FPS/SQS plants. (a) Plants after 5 weeks in the growth chamber. (b, c) Glasshouse‐grown plants after 1 week (b, front), 3 weeks (b, back) or 5 months (c). (d) Shoot apices. (e) Aborted buds. (f–i) Phenotypes of CN (f), N (g), C (h) and WT (i) flowers. FPS, *FARNESYL DIPHOSPHATE SYNTHASE*; SQS, *SQUALENE SYNTHASE*; WT, wild‐type

Regardless of the genome from which transgenes were expressed or levels of Flag‐SQS, expression of *FPS* and *SQS* had a profound effect on leaf and flower development, but CN displayed most severe leaf and flower phenotypes. At the time of transfer to soil, CN leaves were half as long as WT (Figure 2a) and remained small, both after transferring to the glasshouse (Figure 2b) and at the onset of flowering (Figure 2c). In particular, leaves of adult CN plants were shorter, narrower and more curled (Figure 2c). Leaves of N were also initially shorter than WT (Figure 2a,b), but as they aged, leaf morphology more closely resembled that of WT (Figure 2c). A similar phenomenon was observed for C plants, whose pleiotropic phenotypes became less pronounced with age (Figure 2b,c).

Inflorescences of engineered lines were more clustered than those of WT (Figure 2d), and many buds aborted prematurely (Figure 2e); these buds contained tiny, underdeveloped stigmas and desiccated petals and stamens (Figure 2e). In CN flowers, stamens were on long, curved filaments that produced minimal pollen (Figure 2f). By contrast, N and C flowers displayed stamens on straight filaments with anthers that contained noticeably more pollen (Figure 2g,h), although N filaments were generally longer and anthers contained more pollen than C (compare Figure 2g with Figure 2h). Unlike in engineered lines, WT stamens were the same length as stigmas, and anthers produced abundant pollen (Figure 2i).

Notably, because of differences in antibiotic selection when plants were initially grown (WT and N on antibiotic‐free media and CN and C on spectinomycin‐containing media), it is more meaningful to compare CN with C and WT with N. Given this, differences between CN and C are quite striking; despite both expressing high levels of Flag‐SQS, CN had much more severe phenotypes. Similarly, N's phenotypes were as clear as those of CN and C and occurred despite expressing comparatively little Flag‐SQS.

### Evaluation of metabolic profiles of transgenic/transplastomic lines expressing *FPS* and *SQS*

**Figure 3:**
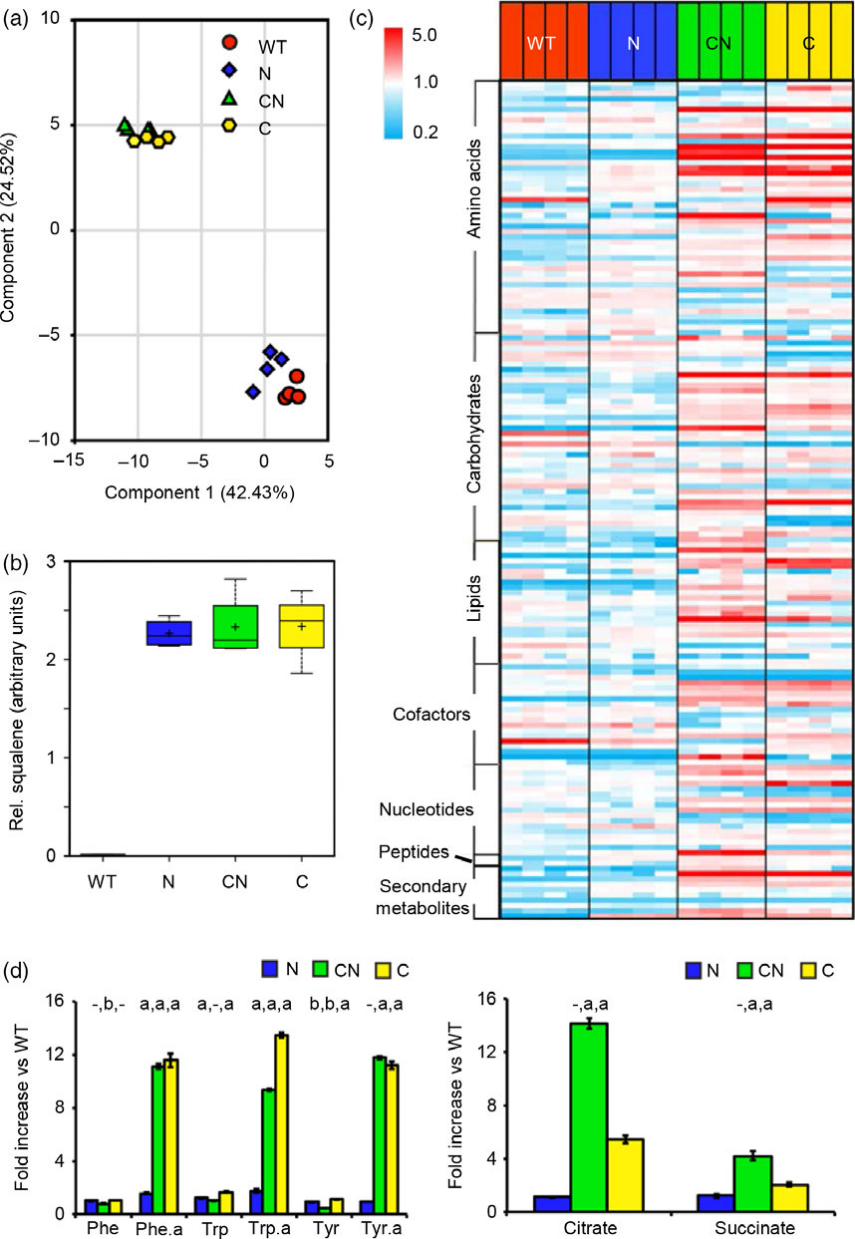
Impact of *FPS*/*SQS* expression on levels of cellular metabolites. (a) PCA showing clustering of quadruplicate samples from each line. (b) Boxplot showing squalene levels. (c) Heat map showing functional categories of metabolites. Each box represents one sample. (d) Graphs showing the accumulation of selected metabolites compared with WT, including aromatic amino acid derivatives (left) and TCA cycle‐related metabolites (right). Error bars indicate SEM. Abbreviations for statistical analysis are clustered in groups of three and are ordered N, CN, C. a, statistically significant increase over WT; b, statistically significant decrease relative to WT; –, difference with WT is not statistically significant. In the left panel, three letter abbreviations are those of amino acids; Phe.a, phenethylamine; Trp.a, tryptamine; Tyr.a, tyramine. FPS, *FARNESYL DIPHOSPHATE SYNTHASE*; PCA, principal component analysis; SQS, *SQUALENE SYNTHASE*; WT, wild‐type.

Although our original intention was to produce transplastomic plants that accumulated squalene, phenotypes of all three lines suggested that more than just squalene levels might be altered. Therefore, we used nodal cutting to grow all lines *in vitro* under identical physiological conditions and harvested the third youngest leaf from plants at an identical developmental stage to analyse global metabolic profiles. All transplastomic plants were from the T0 generation. The metabolomic analysis used gas chromatography/mass spectrometry (GC/MS) and two types of ultra high‐performance liquid chromatography/tandem mass spectrometry (UHPLC/MS/MS), one optimized for acidic species and one optimized for basic species (Clarke *et al*., 2013; Evans *et al*., 2009). Principal component analysis (PCA) of quadruplicate samples showed tight clustering within each line (Figure 3a). PCA also showed that metabolic profiles of CN and C were distinct from N and WT but were almost indistinguishable from each other, whereas N showed a small but clear separation from WT (Figure 3a).

Metabolomic‐based analysis of squalene levels indicated that despite up to 4000‐fold higher Flag‐SQS levels in CN and C than in N (Figure 1d), all three lines accumulated approximately 150‐fold more squalene than WT (Figure 3b). As predicted, however, the abundance of dozens of other metabolites changed statistically significantly in all three engineered lines, and they encompassed multiple functional categories, including amino acids, carbohydrates, nucleotides and lipids (Figure 3c, Table S1). Notably, when a metabolite's abundance was significantly changed in all three lines, the direction of change was generally identical (Figure 3c, Tables 1 and S1).

**Table 1:** List of metabolites whose abundance changes at least 10‐fold compared with wild‐type (WT)

Metabolite	Category	Family	N/WT	CN/WT	C/WT
Squalene	Secondary metabolism	Terpenoids	154.10^a^	158.57^a^	158.86^a^
3‐(2‐piperidinyl)pyridine	Secondary metabolism	Alkaloids	−1.17	32.16^a^	31.82^a^
Pheophorbide A	Cofactors, prosthetic groups, electron carriers	Chlorophyll and haem metabolism	1.00	25.52^a^	2.58
Gluconate	Carbohydrate	Amino sugar and nucleotide sugar	1.55	24.85^a^	10.42^a^
Citrate	Carbohydrate	TCA cycle	1.13	14.16^a^	5.41^a^
Tryptamine	Amino acid	Aromatic amino acid metabolism (PEP derived)	1.76^a^	9.35^a^	13.48^a^
4‐hydroxybutyrate (GHB)	Amino acid	Glutamate family (alpha‐ketoglutarate derived)	−1.75	12.54^a^	−1.50
Tyramine	Amino acid	Aromatic amino acid metabolism (PEP derived)	−1.05	11.78^a^	11.21^a^
Phenethylamine	Amino acid	Aromatic amino acid metabolism (PEP derived)	1.57^a^	11.11^a^	11.55^a^
1‐palmitoyl‐GPI (16:0)	Lipid	Phospholipid	1.01	11.27^a^	3.93^a^

a Statistically significant difference in the accumulation of the indicated metabolite compared with WT.

In both CN and C, approximately 120 metabolites changed significantly (Figure 3c, Table S1), and several accumulated more than 10‐fold (Figure 3c, d, Table 1). Similarly, a transplastomic line in the Petit Havana (PH) background expressing only *SQS* significantly affected the abundance of nearly 100 metabolites (Table S2). Of these, the most notable was nicotine, which accumulated to nearly 11‐fold higher levels than in untransformed plants (Table S2). Among those metabolites that accumulated in plants engineered in the WT 1068 background, three—tryptamine, tyramine and phenethylamine—increased without a corresponding increase in their aromatic amino acid precursors (Figure 3d, Table 1). Two TCA cycle‐associated metabolites, citrate and succinate, accumulated to 15.2‐fold and fourfold higher levels, respectively, in CN than in WT (Figure 3d, Table 1). In C, these metabolites accumulated to 5.4‐fold and twofold higher levels than in WT (Figure 3d, Table 1). We also noted a concomitant reduction in photosynthetic Calvin cycle outputs fructose 6‐phosphate (repressed up to fivefold) and glucose 6‐phosphate (repressed up to 6.25‐fold) (Table S1). In N, the abundance of approximately 65 metabolites changed statistically significantly (Figure 3c, Table S1), but with the exception of squalene (Figure 3b), they changed only two‐ to threefold at most (Figure 3c, Tables 1 and S1).

### The impact of *FPS* and *SQS* expression on the transcription of nuclear genes

**Figure 4:**
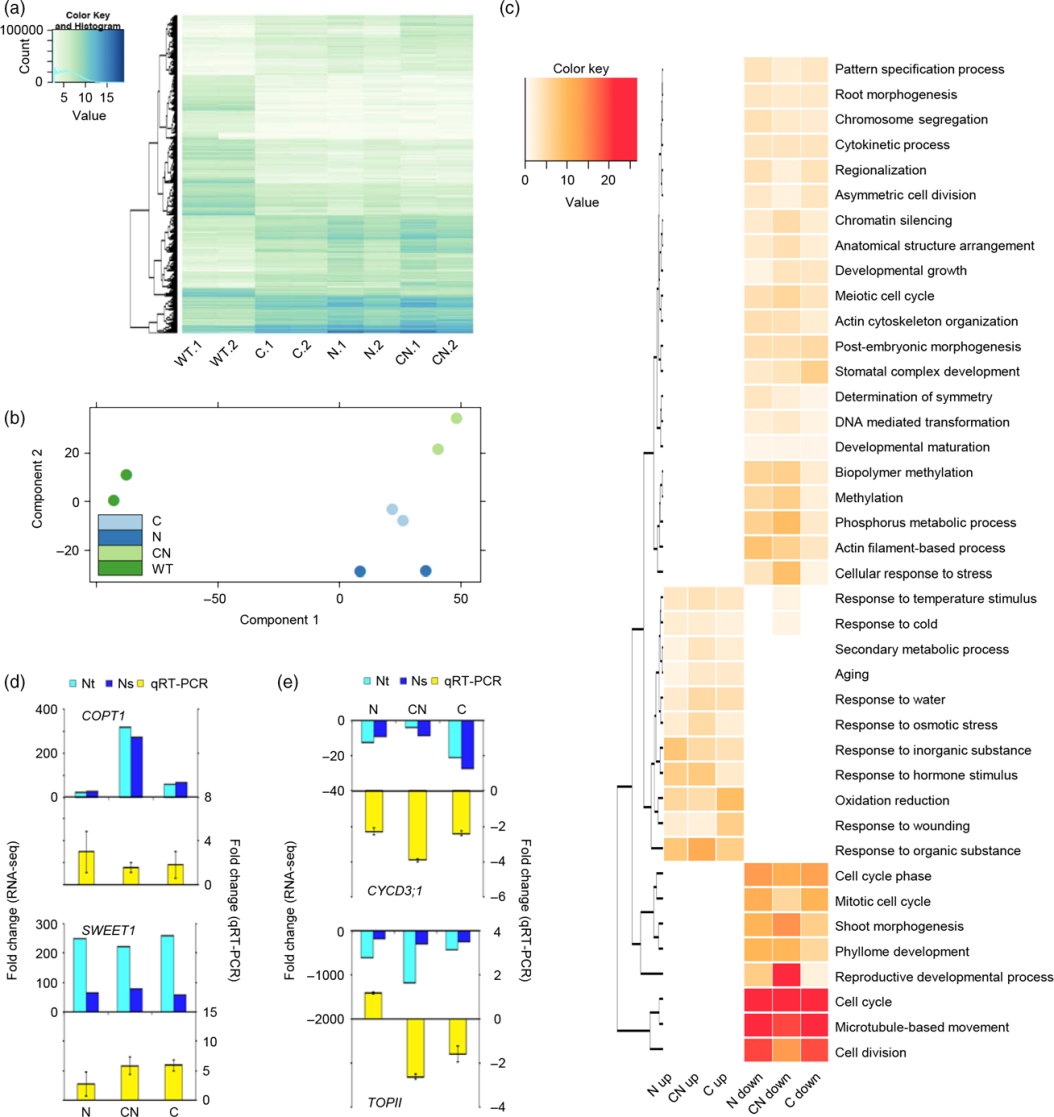
RNA‐seq analysis of FPS/SQS plants. (a) Hierarchical clustering analysis and (b) PCA of duplicate samples. (c) Heat map showing overrepresented biological processes for up‐ and down‐regulated transcripts in each line. Indicated values are –log_10_ of the Benjamini adjusted p‐value. (d, e) RNA‐seq data for transcripts derived from *Nicotiana tomentosiformis* (Nt, cyan) and *Nicotiana sylvestris* (Ns, blue) genomes (upper panels, primary *y*‐axis) and qRT‐PCR confirmation of transcript levels (lower panels, yellow, secondary *y*‐axis) for transcripts that are induced (d) or repressed (e) relative to WT. In lower panels, error bars represent mean ± SEM of two biological replicates with three technical replicates each. FPS, *FARNESYL DIPHOSPHATE SYNTHASE*; PCA, principal component analysis; SQS, *SQUALENE SYNTHASE*; WT, wild‐type.

The metabolic changes and physiological phenotypes in all three lines prompted us to investigate transcriptional mechanisms that underlie these changes. Therefore, we performed poly(A)‐selected RNA sequencing (RNA‐seq) on leaf tissue from plants of each line. Leaf material was distinct from that used for the metabolomic analysis but was grown in an identical manner and harvested at an identical developmental stage, with transplastomic plants in the T0 generation. Importantly, poly(A) selection enriches for nuclear‐encoded transcripts and depletes chloroplast transcripts, which lack an extended poly(A) tail. High‐throughput sequencing reads were mapped to the tobacco genome, and transcripts were identified and quantified as previously described (Sierro *et al*., 2014). Hierarchical clustering analysis of expression counts for predicted transcripts demonstrated that expression patterns of each replicate for the three different transgenic lines were similar (Figure 4a). PCA showed close clustering within lines (Figure 4b). Unlike the metabolomic analysis, which showed N clustering more closely with WT and transplastomic CN and C lines clustering together (Figure 3a), transcriptomic analysis showed that overall gene expression profiles of each line were fairly separate. However, transcriptomes of engineered lines were more similar to each other than to WT (Figure 4b).

To annotate predicted transcripts, we first identified likely open reading frames (ORFs) and then used BLASTP with the NCBI *Nicotiana* database. This analysis led to many transcripts being assigned ‘unknown’ or ‘uncharacterized’ annotations or given no annotation. Additionally, multiple transcripts were often annotated to the same gene. Therefore, to increase the number of uniquely annotated ORFs, we used BLASTP with the TAIR10 version of the *Arabidopsis* transcriptome and assigned *Arabidopsis*‐based annotations to the predicted transcripts. This analysis permitted more detailed functional categorization of each transcript and allowed the assignment of the most closely matched annotation to each transcript model.

At the 99.9% confidence level, more than twice as many transcripts were differentially expressed (DE) in CN: 19 076 compared with 7784 in N and 5224 in C. First, we found a 4300‐fold increase in *FPS* expression and a 7200‐fold increase in *SQS* expression in N compared with WT. In CN, *FPS* expression increased 3600‐fold and nuclear transgenic *SQS* expression increased 1300‐fold relative to WT. Next, we determined which transcripts were most highly changed in each line compared with WT, ranked them by overall fold change independent of the line(s), then identified the fold change in other two lines. Despite identifying DE transcripts in a line‐independent manner, the majority of most highly induced (Table 2) and most highly repressed transcripts (Table 3) were common to all three lines and changed in the same direction. Strikingly, nearly half of most highly induced transcripts (9/20) were predicted to encode transporters of varying functions, including transporters for sucrose (SWEETs), phosphate, metals and auxin. In addition, transcripts with predicted roles in growth and development, metabolism and defence were induced. Consistent with these annotations, Gene Ontology (GO) analysis of biological process‐related terms showed enrichment for terms related to stress and stimulus responses (Figure 4c). We also observed enrichment for transcripts involved in secondary metabolism (Figure 4c). GO analysis of cellular component‐ and molecular function‐specific terms showed slight enrichment for photosynthesis‐related components and processes (Figures S2 and S3).

**Table 2:** Top 20 overall most highly induced transcripts and their expression in other two transgenic/transplastomic lines, grouped by functional category. When possible, each locus includes an annotation for *Nicotiana tomentosiformis* (Nt) and an annotation for *Nicotiana sylvestris* (Ns). Values without asterisks indicate 99.9% confidence. Cells without values were not significantly changed for the indicated transcript in the indicated line

Rank	NCBI gene ID/At number	*Nicotiana*/*Arabidopsis* annotation	N/Wild‐type (WT)	CN/WT	C/WT
Transporters					
1	LOC104087257/At4g12510	Nt *14 kDa PROLINE‐RICH PROTEIN DC2.15*‐like/2S albumin superfamily protein	806.987	940.608	172.152
	LOC104242032/At4g12510	Ns *14 kDa PROLINE‐RICH PROTEIN DC2.15*‐like/2S albumin superfamily protein	73.826	56.626	18.003
4	LOC104088673/At3g48740	Nt *N3 SUGAR TRANSPORTER*‐like/*SWEET11*	87.235	201.150	65.779
	LOC104243017/At3g48740	Ns *SWEET12*‐like/SWEET11	148.741	327.890	72.298
5	LOC104115159/At3g54700	Nt probable *INORGANIC PHOSPHATE 1‐7*/*PHT1;7*	11.423^a^		15.762
	LOC104216800/At3g54700	Ns probable *INORGANIC PHOSPHATE TRANSPORTER 1‐2*/*PHT1;7*	10.502^a^	323.984	299.250
6	LOC104086110/At5g59030	Nt *COPPER TRANSPORTER 1*‐like/*COPT1*	20.421	318.867	55.805
	LOC104244096/At5g59030	Ns *COPPER TRANSPORTER 1*‐like/*COPT1*	23.937	271.475	68.523
8	LOC104084825/At5g16530	Nt putative *AUXIN EFFLUX CARRIER COMPONENT 8*/*PIN5*		299.984	48.512
	LOC104249472/At5g16530	Ns putative *AUXIN EFFLUX CARRIER COMPONENT 8*/*PIN5*		201.077	32.503
10	LOC104086686/At1g21460	Nt *SWEET1*‐like/*SWEET1*	248.285	220.603	259.497
	LOC104225662/At1g21460	Ns *SWEET1*‐like/*SWEET1*	63.264	79.362	57.860
12	LOC104101946/At5g59520	Nt *ZINC TRANSPORTER 2*/*ZIP2*		32.226	
	LOC104211888/At5g59520	Ns *ZINC TRANSPORTER 2*/*ZIP2*	13.010	249.781	15.498
14	LOC104096904/At5g53190	Nt bidirectional sugar transporter *SWEET3b*/*SWEET3*		182.521	140.590
	LOC104241809/At5g53190	Ns bidirectional sugar transporter *SWEET3*/*SWEET3*		236.357	107.210
17	LOC104118881/At5g47560	Nt *TONOPLAST DICARBOXYLATE TRANSPORTER*/*TDT*	37.066	9.426	
	LOC104242854/At5g47560	Ns *TONOPLAST DICARBOXYLATE TRANSPORTER*/*TDT*	221.581	33.801	
Growth and development					
2	LOC104105402/At5g62040	Nt *CEN*‐like 1/*BFT*		538.996	
	LOC104229471/At5g62040	Ns *CEN*‐like 1/*BFT*		431.913	14.363
3	LOC104089336/At5g10625	Nt *FLOWERING PROMOTING FACTOR 1*‐like 3/*FPF1*‐like	115.961	356.184	16.807
	LOC104232534/At5g10625	Ns *FLOWERING PROMOTING FACTOR 1*‐like 3/*FPF1*‐like		440.531	12.179^a^
7	LOC104104199/At5g06720	Nt *PEROXIDASE 15*/*PA2*	20.544	312.576	80.377
	LOC104217681/At5g06720	Ns *LIGNON‐FORMING ANIONIC PEROXIDASE*‐like/*PA2*		81.758	
13	LOC104243032/At4g25820	Ns *XYLOGLUCAN ENDOTRANSGLUCOSYLASE/HYDROLASE 24*‐like/*XTR9*	197.748	242.975	69.681
Secondary metabolism					
9	LOC104099208/At4g14090	Nt *CROCETIN GLUCOSYLTRANSFERASE*/*ANTHOCYANIDIN‐5‐O‐GLUCOSYLTRANSFERASE*	94.367	272.367	18.904
	LOC104227958/At4g14090	Ns *CROCETIN GLUCOSYLTRANSFERASE*/*ANTHOCYANIDIN‐5‐O‐GLUCOSYLTRANSFERASE*	9.567	12.620	4.611
15	LOC104104691/At4g19170	Nt *CAROTENOID CLEAVAGE DIOXYGENASE 4*/*NCED4*	131.189	101.045	234.293
19	LOC104107213/At4g15480	Nt *CINNAMATE BETA‐D‐GLUCOSYLTRANSFERASE*/*UGT84A1*	214.326	200.087	142.143
Hormone					
11	LOC104088764/At1g75750	Nt *GIBBERELLIN‐REGULATED 1*‐like/*GASA1*	58.475	256.662	11.149^a^
Primary metabolism					
16	LOC104096882/At5g48850	Nt uncharacterized/*SDI1*	38.021	227.743	
Cytoskeleton					
18	LOC104116945/At1g70810	Nt probable ADP‐ribosylation factor GTPase‐activating protein *AGD11*/*CAR7*	9.630	91.183	12.693
	LOC104249050/At1g70810	Ns probable ADP‐ribosylation factor GTPase‐activating protein *AGD11*/*CAR7*	52.144	216.566	32.270
Defence					
20	LOC104103886/At2g38870	Nt *PROTEINASE INHIBITOR I‐B*/*PR6*‐like	79.968	100.779	23.172
	LOC104231145/At2g38870	Ns *PROTEINASE INHIBITOR I‐A*/*PR6*‐like	131.969	210.761	35.884

a Value reported at the 99.0% confidence level.

**Table 3:** Top 20 overall most highly repressed transcripts and their expression in other two transgenic/transplastomic lines, grouped by functional category. When possible, each locus includes an annotation for *Nicotiana tomentosiformis* (Nt) and an annotation for *Nicotiana sylvestris* (Ns)

Rank	NCBI gene ID/At number	*Nicotiana* annotation/*Arabidopsis* annotation	N/Wild‐type (WT)	CN/WT	C/WT
Cell cycle					
1	LOC10409419/At1g30690	Nt *PATELLIN‐4*/*Sec14*‐like	−816.166	−263.187	−2262.789
	LOC10421949/At1g30690	Ns *PATELLIN‐4*/*Sec14*‐like	−493.844	−746.213	−666.637
7	LOC10408601/At5g06150	Nt *CYCLIN S13‐7*‐like/*CYCB1;2*	−726.831	−364.512	−559.138
	LOC10423757/At5g06150	Ns *CYCLIN S13‐7*‐like/*CYCB1;2*	−1052.573	−524.124	−1325.489
10	LOC10421173/At4g11080	Ns *HIGH MOBILITY GROUP B 6*‐like/*3XHMG‐BOX1*	−531.236	−317.492	−1124.502
11	LOC10424297/At1g44110	Ns *CYCLIN‐A1‐4*/*CYCA1;1*	−257.417	−401.190	−989.323
12	LOC10409411/At3g20150	Nt kinesin‐like *KIN12A*/kinesin family protein	−732.380	−361.282	−918.263
	LOC10424399/At3g20150	Ns kinesin‐like *KIN12A*/kinesin family protein	−368.772	−322.527	−453.958
13	LOC10411222/At1g03780	Nt *TPX2*‐like/*TPX2*	−584.023	−504.647	−308.380
	LOC10421712/At1g03780	Ns *TPX2*‐like/*TPX2*	−915.733	−790.737	−694.173
15	LOC10410080/At2g26760	Nt *CYCLIN S13‐7*‐like/*CYCB1;4*	−682.727	−593.531	−850.024
	LOC10422394/At2g26760	Ns *CYCLIN S13‐7*‐like/*CYCB1;4*	−248.516	−119.077	−306.997
16	LOC10409883/At1g08560	Nt *KNOLLE*/*KNOLLE*	−654.447	−125.074	−298.701
	LOC10422292/At1g08560	Ns *KNOLLE*/*KNOLLE*	−647.369	−163.412	−847.873
Cell wall/cuticle					
3	LOC10411994/At4g22010	Nt *L‐ASCORBATE OXIDASE* homologue/*SKS4*	−459.175	−660.733	−594.408
	LOC10423150/At4g22010	Ns *L‐ASCORBATE OXIDASE* homologue*/SKS4*	−481.000	−1001.534	−1473.924
6	LOC10410009/At5g47500	Nt probable *PECTINESTERASE 68*/*PME5*	−642.662	−370.989	−1355.422
	LOC10421908/At5g47500	Ns probable *PECTINESTERASE 68*/*PME5*	−345.366	−518.392	−831.930
19	LOC10409611/At1g02205	Nt *ECERIFERUM 1*‐like/*CER1*	−38.939	−91.297	−78.052
	LOC10424030/At1g02205	Ns *ECERIFERUM 1*‐like/*CER1*	−199.464	−546.898	−799.481
Kinase					
4	LOC10411606/At3g51740	Nt probably inactive leucine‐rich repeat receptor‐like protein kinase *IMK2*/*IMK2*	−1446.626	−764.462	−457.671
	LOC10423705/At3g51740	Ns probable leucine‐rich repeat receptor‐like protein kinase *IMK3*/*IMK3*	−305.037	−265.833	−219.477
17	LOC10409992/At5g43020	Nt probable inactive receptor kinase At5g67200/LRR kinase family	−168.644	−572.847	−841.587
	LOC10423543/At5g43020	Ns probable inactive receptor kinase At5g67200/LRR kinase family	−32.928	−96.819	−44.635
Miscellaneous					
18	LOC10409693/At4g31840	Nt *EARLY NODULIN‐LIKE 1*/*ENODL15*	−395.968	−118.288	−821.479
	LOC10421187/At4g31840	Ns *EARLY NODULIN‐LIKE 3*/*ENODL15*	−343.122	−120.464	−623.076
20	LOC10412083/At5g23420	Nt *HIGH MOBILITY GROUP B 7*‐like/*HMGB6*	−500.882	−134.517	−476.056
	LOC10424371/At5g23420	Ns *HIGH MOBILITY GROUP B 7*‐like/*HMGB6*	−262.125	−222.445	−790.240
Lipid metabolism					
2	LOC10412153/At5g33370	Nt GDSL esterase/lipase At5g33370‐like/GDSL‐motif esterase/lipase	−546.326	−2106.034	−1221.907
	LOC10422041/At5g33370	Ns GDSL esterase/lipase At5g33370‐like/GDSL‐motif esterase/lipase	−79.807	−93.596	−224.898
Secondary metabolism					
5	LOC10408627/At5g66230	Nt uncharacterized/chalcone‐flavanone isomerase	−828.169	−136.904	−490.657
	LOC10421950/At5g66230	Ns uncharacterized/chalcone‐flavanone isomerase	−686.380	−310.377	−1419.234
Growth and development					
8	LOC10409319/At2g42840	Nt *PROTODERMAL FACTOR 1*‐like/*PDF1*	−1003.212	−869.240	−1255.300
	LOC10422232/At2g42840	Ns *PROTODERMAL FACTOR 1*‐like/*PDF1*	−486.103	−145.233	−302.012
DNA remodelling					
9	LOC10412078/At3g23890	Nt *DNA TOPOISOMERASE II*‐like/*TOPII*	−615.688	−1172.774	−442.749
	LOC10424672/At3g23890	Ns *DNA TOPOISOMERASE II*‐like/*TOPII*	−189.431	−287.318	−247.807
Cytoskeleton					
14	LOC10408502/At1g03470	Nt uncharacterized/*KIP1*‐like	−688.519	−235.486	−866.163
	LOC10422085/At1g03470	Ns uncharacterized/*KIP1*‐like	−306.668	−252.734	−287.424

For a few transcripts, for example *SWEET1* (transcript 10), expression was induced similarly across all three lines (Table 2). However, for many other induced transcripts, although the largest change in expression differed, the greatest induction occurred most often in CN. For example, *COPT1* (transcript 6) and *PIN5* (transcript 8) were both induced approximately 300‐fold in CN, whereas in N, *COPT1* was induced only 20‐fold and *PIN5* was not significantly changed; and in C, *COPT1* was induced 70‐fold and *PIN5* was induced 50‐fold. By contrast, only one transcript was most highly induced each in C (*Nicotiana tomentosiformis NCED4*, transcript 15) and N (*Nicotiana sylvestris TONOPLAST DICARBOXYLATE TRANSPORTER*, transcript 17), although the *N. tomentosiformis 14‐kDa protein* (a lipid transporter, transcript 1) was also induced very highly in N—more than 800‐fold (Table 2). Additionally, a subset of transcripts was induced to a similar level in both N and CN, including the *N. tomentosiformis 14‐kDa protein* (transcript 1, 900‐fold in CN and 800‐fold in N) and both *N. tomentosiformis* and *N. sylvestris PR6‐*like proteinase inhibitor (transcript 20, 100–200‐fold in CN and ~100‐fold in N) (Table 2).

Table 3 lists the 20 most highly repressed transcripts, with more than half (12/20) predicted to function in the cell cycle, cell wall remodelling and DNA remodelling, and additional functional categories included kinases, metabolism and growth and development. GO analysis for ‘biological process’‐specific terms confirmed substantial enrichment among repressed transcripts for those with predicted functions in the cell cycle (Figure 4c). GO analysis for ‘cellular component’‐related terms showed enrichment for cell wall, chromosomes and cytoskeletal components among down‐regulated transcripts (Figure S2), and GO analysis for ‘molecular function’‐related terms showed enrichment for transcripts predicted to be involved in binding nucleosides and cytoskeletal proteins (Figure S3).

Without exception, each of the most highly repressed transcripts were repressed across all three lines, and there were some—for example the *N. tomentosiformis TPX2*‐like gene (transcript 13) and the *N. sylvestris KIP1*‐family gene (transcript 14)—whose expression was comparable in all three lines. However, there were other transcripts for which the magnitude of change did vary. Strongest repression was often found in C, as in the case of the *N. tomentosiformis PATELLIN‐4* (transcript 1, repressed more than 2000‐fold) and the *N. sylvestris HMG PROTEIN6* (transcript 10, repressed 1000‐fold). In other instances, as for a transcript annotated to a *N. tomentosiformis* GDSL‐motif esterase/lipase (transcript 2) and for a *N. tomentosiformis DNA TOPOISOMERASE II* (transcript 9), CN clearly showed the strongest repression: 2000‐fold and 1100‐fold, respectively. CN also showed a unique enrichment for down‐regulated transcripts predicted to be involved in reproductive development (Figure 4c). The N line also had a large effect on the expression of certain transcripts, most notably that for the *N. tomentosiformis* IMK2 kinase (transcript 4, repressed 1400‐fold). Two other transcripts, *N. sylvestris CYCLIN B1;2* and *N. tomentosiformis PDF1* (transcripts 7 and 8, respectively) were also repressed more than 1000‐fold in N (Table 3). Interestingly, in cases in which a transcript was most highly repressed in two lines, the two with the strongest effect tended to be N and C (e.g. for *N. sylvestris CYCLIN B1;2* and *N. tomentosiformis* kinesin *KIN12A*).

To further confirm the validity of RNA‐seq data, we performed qRT‐PCR for select induced and repressed transcripts using biologically independent samples at an identical developmental stage. All transcripts predicted to be induced by RNA‐seq were verified to be induced by qRT‐PCR (Figure 4d), and with one exception (*TOPII* in N), transcripts predicted by RNA‐seq to be repressed were also repressed relative to WT in qRT‐PCR (Figure 4e). Plotting the log_2_ fold change of each transcript using qRT‐PCR versus RNA‐seq showed a good correlation between results from both methods, with R values ranging from 0.690 to 0.899 (Figure S4).

## Discussion

To date, few studies on plant metabolic engineering have looked beyond the pathway of interest to assess unanticipated consequences, and no report has compared metabolomic and transcriptomic effects of expressing transgenes from different organellar genomes. Here, we demonstrate that expressing chloroplast‐targeted FPS and/or SQS from the nuclear genome (N line), the chloroplast genome (C line) or both (CN line) has broad off‐target effects on the metabolome and transcriptome. However, because many of the same metabolites and transcripts are changed in a similar direction across all three lines, *FPS* and *SQS* expression may cause broad but somewhat predictable off‐target effects.

Because the chloroplast genome promotes higher transgene expression than the nuclear genome (Verma and Daniell, 2007), one goal of this study was to increase squalene production by transplastomically expressing codon‐optimized *FPS* and/or *SQS*. However, neither transplastomic line accumulated significantly more squalene than N (Figure 3b, Tables 1 and S1), suggesting a substrate or storage limit. Consistent with this, in the background of PH, which has fewer glandular trichomes than 1068 (Wu *et al*., 2012), we could generate plants expressing *SQS* only; transforming PH chloroplasts with pLD‐FPS‐SQS yielded shoots that did not survive, and similar problems were encountered using nuclear transformation constructs that targeted FPS and SQS to chloroplasts. Inadequate trichomes (sinks) in PH may have caused this, perhaps through feedback inhibition. Indeed, the 2‐*C*‐methyl‐d‐erythritol 4‐phosphate (MEP) pathway of terpene biosynthesis is under feedback inhibition by its own products, dimethylallyl diphosphate (DMAPP) and isopentenyl diphosphate (IPP) (Banerjee *et al*., 2013), and IPP is the substrate of FPS. One strategy to increase squalene could be to increase sink capacity by sequestering squalene in storage compartments such as lipid droplets (Wang *et al*., 2015) or additional trichomes (Lange, 2015). Trichome density could be increased *via* transgenic expression of β‐glucosidase (Jin *et al*., 2011), which improves artemisinin yields in transgenic *Artemisia annua* (Singh *et al*., 2015).

Among the 65–120 metabolites changed across engineered lines, in transplastomic lines, we noted an increase in TCA cycle‐related metabolites, including citrate and succinate (Figure 3d), and a reduction in fructose 6‐phosphate and glucose 6‐phosphate (Table S1). Fructose 6‐phosphate and glucose 6‐phosphate are both photosynthetic outputs from the Calvin cycle and are synthesized from glyceraldehyde 3‐phosphate, a three‐carbon Calvin cycle output. In chloroplasts, squalene biosynthesis is initiated by the conversion of glyceraldehyde 3‐phosphate and pyruvate into 1‐deoxy‐d‐xylulose‐5‐phosphate in a committed step catalysed by 1‐deoxy‐d‐xylulose‐5‐phosphate synthase. Therefore, the 150‐fold increase in squalene levels and the decrease in fructose 6‐phosphate and glucose 6‐phosphate biosynthesis observed in engineered lines suggest that glyceraldehyde 3‐phosphate is repartitioned from the synthesis of sugars into the production of terpenes (Wang *et al*., 2015).

The chloroplast amino acid pool is limited (Bally *et al*., 2009), and chloroplast transgene products are produced at the expense of resident proteins such as Rubisco (Bally *et al*., 2011; Ruhlman *et al*., 2010). Therefore, given the accumulation of Flag‐SQS in transplastomic lines (Figure 1d), it is not surprising that amino acid metabolism was altered in CN and C (Figure 3c and d, Table 1). Decarboxylated aromatic amino acids accumulated to at least eightfold higher levels in CN and C than in WT without an increase in their corresponding precursors (Figure 3d, Table 1). This result demonstrates that transplastomic lines are not impaired in amino acid biosynthesis and suggests the diversion of carbon into squalene biosynthesis, leading to an imbalance between carbon and nitrogen that explains the accumulation of these amino acid derivatives. Additionally, the abundance of various nucleotides was affected (Figure 3c; Table S1), possibly due to large quantities of *FPS* and/or *SQS* mRNA.

Transcriptomic analysis revealed thousands of DE transcripts between each line and WT (Figure 4a). Induction of transporter‐related transcripts (Table 2) may reflect the need to move additional metabolites from sites of synthesis to sites of use or storage. Additionally, highly repressed cell cycle transcripts (Table 3, Figure 4c) correlated well with growth phenotypes (Figure 2c). Importantly, all most highly DE transcripts were changed in the same direction across all three lines (Tables 2 and 3, Figure 4d,e), regardless of the compartment(s) from which transgenes were expressed.

We also observed differences in transcriptomes that varied with the compartment(s) from which transgenes were expressed, including the number and magnitude of change of DE transcripts. Importantly, transcriptomic data also showed no correlation between transgene expression and the effect on the expression of nuclear‐encoded genes; the N line expressed up to 4000‐fold less Flag‐SQS than transplastomic lines (Figure 1d) but affected more nuclear‐encoded transcripts than C, and it had the strongest influence on the expression of certain transcripts (Tables 2 and 3). Additionally, when a transcript was most strongly affected in two lines, one of those lines was generally N. Therefore, alterations in gene expression are likely the result of metabolites and not transgene products. Indeed, levels of Flag‐SQS did not vary much between transplastomic lines, but the effect on nuclear‐encoded genes was quite significant.

The number of DE transcripts reported here is considerably greater than those reported in previous studies (Ricroch *et al*., 2011). For example, transgenic rice expressing choline oxidase showed only 165 DE transcripts between engineered and parental lines (Kathuria *et al*., 2009). In transgenic potatoes with elevated or knocked down expression of *sucrose synthase*, 50 and 357 DE genes were found, respectively (Baroja‐Fernández *et al*., 2009). In transgenic rice expressing *anthranilate synthase*, after correcting for variation between samples, only 22 genes met authors’ criteria for differential expression (Dubouzet *et al*., 2007). Compared with DNA microarrays, the greater sensitivity of RNA‐seq may facilitate the detection of far more transcriptomic changes (Wang *et al*., 2009), and transgene expression from different cellular compartments likely also increased the number of DE transcripts (discussed below).

One potential explanation for strong phenotypes observed in CN is disparate levels of FPS and SQS transgene products. Transplastomic CN and C lines accumulate at least 2000‐fold more Flag‐SQS than N (Figure 1d), and expression of *FPS* from the nuclear genome alone may not produce enough transgene product to match levels of SQS in CN (Figure 1c). Therefore, phenotypes observed in CN may be mitigated by incorporating *FPS* into the chloroplast genome. Similarly, in transplastomic PH plants expressing only *SQS*, the abundance of dozens of metabolites changed by >10‐fold (Table S2). Notably, one of these metabolites was nicotine, which accumulated to nearly 11‐fold higher levels than in untransformed plants (Table S2), supporting the idea that metabolic imbalance can result in substantial off‐target effects on entirely unrelated pathways.

Because of transgene compartmentalization, transplastomic plants are excellent tools to study retrograde signalling. In CN, because the parental N line only contained approximately 8000 DE transcripts, additional 11 000 DE transcripts may have changed due to retrograde signalling. In the C line, because transgenes and resulting products are totally contained within chloroplasts, all changes in the expression of the more than 5000 nuclear‐encoded transcripts must be the result of retrograde signalling.

Although our data do not permit us to identify the retrograde signal, it is likely not squalene; otherwise, similar squalene levels (Figure 3b) would cause similar changes in gene expression. However, not only the number of DE transcripts differed, but also their expression levels (Tables 2 and 3). The signal may therefore be a different metabolite or metabolites, perhaps one affected by the imbalance between SQS and FPS levels in CN (Figure 3c, Table S1), but investigation of this possibility is beyond the scope of this study. Interestingly, an upstream molecule in the MEP pathway, methylerythritol cyclodiphosphate (MEcPP), acts as a retrograde signalling molecule under high light and wounding; these stresses induce accumulation of MEcPP to ~two‐ to threefold higher levels than in controls (Xiao *et al*., 2012). Altered flux through the MEP pathway may change the abundance of MEcPP, thereby causing at least a subset of observed transcriptomic changes in engineered plants. The effect of *FPS* and *SQS* expression could extend downstream of MEcPP and parallel to farnesyl diphosphate (FPP) and/or squalene, and these metabolites may also serve as retrograde signals. Both IPP and DMAPP are precursors for the synthesis of carotenoids such as β‐carotene (Nisar *et al*., 2015), and a volatile β‐carotene derivative, β‐cyclocitral, has been proposed as a stress‐induced retrograde signalling molecule (Ramel *et al*., 2012). Increased demand for IPP by FPS or for FPP by SQS may affect carotenoid metabolism, and, consequently, β‐cyclocitral levels, causing altered retrograde signalling. Although none of these metabolites were significantly changed between WT and engineered lines in our analysis, it does not exclude the possibility that the abundance of one or more of them was altered, leading to changes in phenotype and gene expression. If such a difference had occurred, small changes needed to induce signalling (e.g. for MEcPP) and differences in detection and analytical methods may have excluded these metabolites from analysis, especially if differences were statistically insignificant.

Gene products have also been suggested to act as highly specific retrograde signalling molecules, and transgenes engineered *via* the chloroplast genome that are unrelated to metabolism can influence the expression of nuclear genes. For example, chloroplast expression of *Arabidopsis TIC40* or γ‐tocopherol methyltransferase has been shown to promote massive proliferation of the chloroplast inner membrane and the up‐regulation of associated nuclear‐encoded inner membrane proteins without affecting nuclear‐encoded proteins that are targeted to the outer or thylakoid membranes (Jin and Daniell, 2014; Singh *et al*., 2008). Additionally, proteins have been shown to be released from intact chloroplasts (Kwon *et al*., 2013b), providing yet another observation in support of the idea that proteins themselves can be retrograde signals. Therefore, the retrograde signal(s) acting in this case could be a metabolite or a gene product.

Previous studies on genetically modified plants have reported that environmental factors and cultivar‐specific differences play larger roles in altering the transcriptome and metabolome than does transgene expression (Baker *et al*., 2006; Clarke *et al*., 2013; Kogel *et al*., 2010; Ricroch *et al*., 2011). *FPS* and *SQS* expression may cause clear transcriptomic, metabolomic and phenotypic changes because they are metabolism related. For example, transplastomic tobacco expressing twelve genes for the biosynthesis of artemisinic acid (AA) accumulated only approximately 0.1 mg/g fresh weight (FW) AA, but growth was still reduced (Saxena *et al*., 2014), and nuclear transgenic plants accumulating linalool and nerolidol accumulated 1.5 μg/g FW of these terpenes but still displayed delayed growth (Aharoni *et al*., 2003). These observations may reflect the partitioning of carbon away from biomass and into the desired metabolite (Melis, 2013). By contrast, transgenic/transplastomic expression of genes that are unrelated to metabolism has few to no phenotypic effects and, in the case of transplastomic plants, can often be rescued by adding exogenous nitrogen (Bally *et al*., 2009, 2011; De Cosa *et al*., 2001; Ruhlman *et al*., 2010; Viitanen *et al*., 2004). Therefore, metabolite engineering and not protein engineering likely accounts for these phenotypes.

Using cutting‐edge –omics technology, we show that metabolic engineering *via* the nuclear and/or chloroplast genomes can result in broad off‐target effects in the metabolome and transcriptome. However, these effects may be predictable and can therefore be minimized or exploited. By focusing on global consequences of metabolic engineering rather than simply searching for another individual signalling molecule, our results provide a unique, holistic view of metabolite‐mediated intercompartmental signalling that can be used as a framework for future studies on both metabolic engineering and metabolite‐mediated anterograde and retrograde signalling.

## Experimental procedures

### Vector construction, plant transformation and characterization *via* PCR, Southern blot, northern blot and western blot

The WT 1068 introduction and the N nuclear transgenic line were as previously described (Wu *et al*., 2012). All N plants were homozygous siblings derived from the same transformation event. To construct the vectors used to generate transplastomic lines, DNA sequences encoding Flag‐tagged yeast SQS (GenBank accession NM001179321) and 4xHis‐tagged avian FPS (GenBank accession P08836) were codon‐optimized for enhanced chloroplast expression (Daniell *et al*., 2009) and synthesized by Integrated DNA Technologies (Coralville, IA). The optimized *Flag‐SQS* coding sequence was cloned into the *Nde*I and *Xba*I sites of pLD‐ctv under the control of the *psbA* promoter, 5′‐UTR and 3′‐UTR (Daniell *et al*., 2005). To construct pLD‐FPS‐SQS, an intermediate vector was assembled that contained the *Prrn‐g10/His‐FPS/TrbcL* cassette. This vector was digested with *Sal*I, and the released cassette was ligated into *Sal*I‐digested pLD‐SQS. Both vectors were confirmed by sequencing. Chloroplast transformation of WT with pLD‐FPS‐SQS and of N with pLD‐SQS was performed as described previously (Verma *et al*., 2008). PCR and western blot of putative transplastomic plants were carried out as described (Kwon *et al*., 2013a; Verma *et al*., 2008). Western blot was performed using an anti‐DYKDDDDK antibody (LifeTein, South Plainfield, NJ) at a 1 : 1000 dilution and a horseradish peroxidase‐conjugated anti‐mouse secondary antibody (Southern Biotech, Birmingham, AL, USA) at a 1 : 4000 dilution. Densitometric analysis was carried out using the gel analysis feature of ImageJ, Bethesda, MD, USA. Southern blot was performed using the DIG High Prime DNA Labeling and Detection Kit (Roche Diagnostics, Indianapolis, IN) according to the manufacturer's instructions. In brief, 0.5 μg of total tobacco DNA was digested with *Afl*III or *Hin*dIII and resolved on a 0.8% agarose gel. The DNA was blotted onto a positively charged nylon membrane (Nytran SPC; GE Healthcare, Marlborough, MA). Hybridization with DIG‐labelled probe for the *trnI‐trnA* flanking region (Figure 1a) was conducted at 41 °C in a UVP HB‐1000 hybridizer (UVP LLC, Upland, CA), and signals were detected with CSPD substrate and X‐ray film. Probe for northern blot analysis was synthesized using the DIG High Prime DNA Labeling and Detection Kit with a PCR product containing the *rbcL* 3′‐UTR. Northern blot was performed using total RNA (see below) run on a 0.9% denaturing agarose gel. The RNA was blotted onto a Nytran membrane and hybridized with the probe at 42 °C. Signals were detected as for the Southern blot. For phenotypic analysis of homoplasmic transplastomic lines, data are representative of at least two independent transformation events.

### Metabolome analysis

Samples were harvested from the third leaf of plants of the same developmental stage grown *via* nodal cutting in sterile tissue culture conditions. Harvested tissue was lyophilized in a Genesis lyophilizer (SP Scientific, Warminster, PA), and quadruplicate samples were sent to Metabolon (Durham, NC). Samples were analysed using a platform consisting of GC/MS and two UHPLC/MS/MS analyses, one optimized for acidic species and one for basic species (Clarke *et al*., 2013; Evans *et al*., 2009). Metabolomic data are available in Tables S1 and S2.

### RNA sequencing and analysis and GO analysis

mRNA‐seq was performed as previously described (Elliott *et al*., 2013). Briefly, total RNA was purified from the third youngest leaf of two plants from each line at the same developmental stage grown in sterile tissue culture conditions using a miRNeasy kit (Qiagen, Valencia, CA). Poly(A)+ RNA was isolated using oligo(dT) beads (Thermo Fisher Scientific, Waltham, MA). RNA was fragmented for 7 min using Fragmentation Reagent (Thermo Fisher Scientific). mRNA‐seq libraries were generated using an Illumina mRNA‐seq kit (Illumina, San Diego, CA). Sequencing was performed at the University of Pennsylvania Next Generation Sequencing Core. Reads were trimmed with Cutadapt and mapped to the tobacco genome with Tophat2 (Kim *et al*., 2013; Martin, 2011; Sierro *et al*., 2014; Trapnell *et al*., 2009). Cufflinks was used to predict transcripts (Trapnell *et al*., 2010), HTSeq was used to quantify expression of each transcript (Anders *et al*., 2015), and DESeq2 was used to perform differential expression analysis and data normalization (Love *et al*., 2014). We used TransDecoder (Haas *et al*., 2013) to identify ORFs within predicted transcripts and annotated them to TAIR10 proteins. RNA‐seq data have been deposited in GEO under accession number GSE74103.

To perform GO analysis, transcripts were separated into up‐ and down‐regulated and then merged with identical TAIR10 annotations. These gene lists were used as input for the DAVID functional annotation tool (Huang *et al*., 2009). Heat maps were generated using only level 2 and 3 terms with Benjamini adjusted p‐values < 0.05.

### Total RNA extraction, cDNA synthesis and qRT‐PCR

Total RNA was isolated from the third youngest leaf of two plants per line grown in sterile tissue culture conditions using a PureLink RNA Mini Kit (Thermo Fisher Scientific) with on‐column DNase treatment. Three micrograms of total RNA was reverse‐transcribed using random hexamers and a Reverse Transcription System (Promega, Madison, WI) according to the manufacturer's recommendations. The cDNA was diluted 50‐fold in nuclease‐free water, and 6.5 μL of diluted cDNA was used as template for qRT‐PCRs.

qRT‐PCR was performed in a volume of 20 μL using Power SYBR Green PCR master mix (Thermo Fisher Scientific). Primers used for RT‐PCR analysis are listed in Table S3. Reactions were run on a StepOnePlus Real‐Time PCR system (Thermo Fisher Scientific). Calculations were performed using the 2^ΔΔCt^ method and normalized to *ACT* and *EF1a*.

## Author contributions

E.K.P. performed the research and analysed/interpreted data in Figures 1c and 2, 3, 4, Tables 1, 2, 3 and the supplementary data and wrote this manuscript. J.S. contributed data in Figure 1b,d and related text in Results and Methods sections. I.M.S., S.J.G. and B.D.G. analysed/interpreted data in Figure 4, Tables 2 and 3 and Figures S2–S4 and contributed related text in Results and Materials and methods sections. J.S.Y. analysed/interpreted data and contributed text in the Introduction and Discussion sections. H.D. conceived and designed the study, analysed/interpreted data and wrote this manuscript.

## Conflict of interest

H.D. is an inventor in several U.S. and international patents related to chloroplast transformation technology.

## Supporting information


**Figure S1** PCR analysis of transgene integration in transplastomic plants.

**Figure S2** GO analysis for ‘cellular component’‐related terms.

**Figure S3** GO analysis for ‘molecular function’‐related terms.

**Figure S4** Plot of log_2_ fold changes for selected transcripts as determined by qRT‐PCR versus RNA‐seq.

**Table S1** Metabolites with significantly changed abundance in each line relative to WT.

**Table S2** List of all metabolites whose abundance changes significantly and by at least twofold in PH‐SQS compared with PH.

**Table S3** List of qRT‐PCR primers used in this study.
