# Supplementary figures and images for: Terpene metabolic engineering via nuclear or chloroplast genomes profoundly and globally impacts off‐target pathways through metabolite signalling
Source: Plant Biotechnol J. 2016 Mar 8;14(9):1862–75. doi: 10.1111/pbi.12548 (PMC4980996; doi:10.1111/pbi.12548)

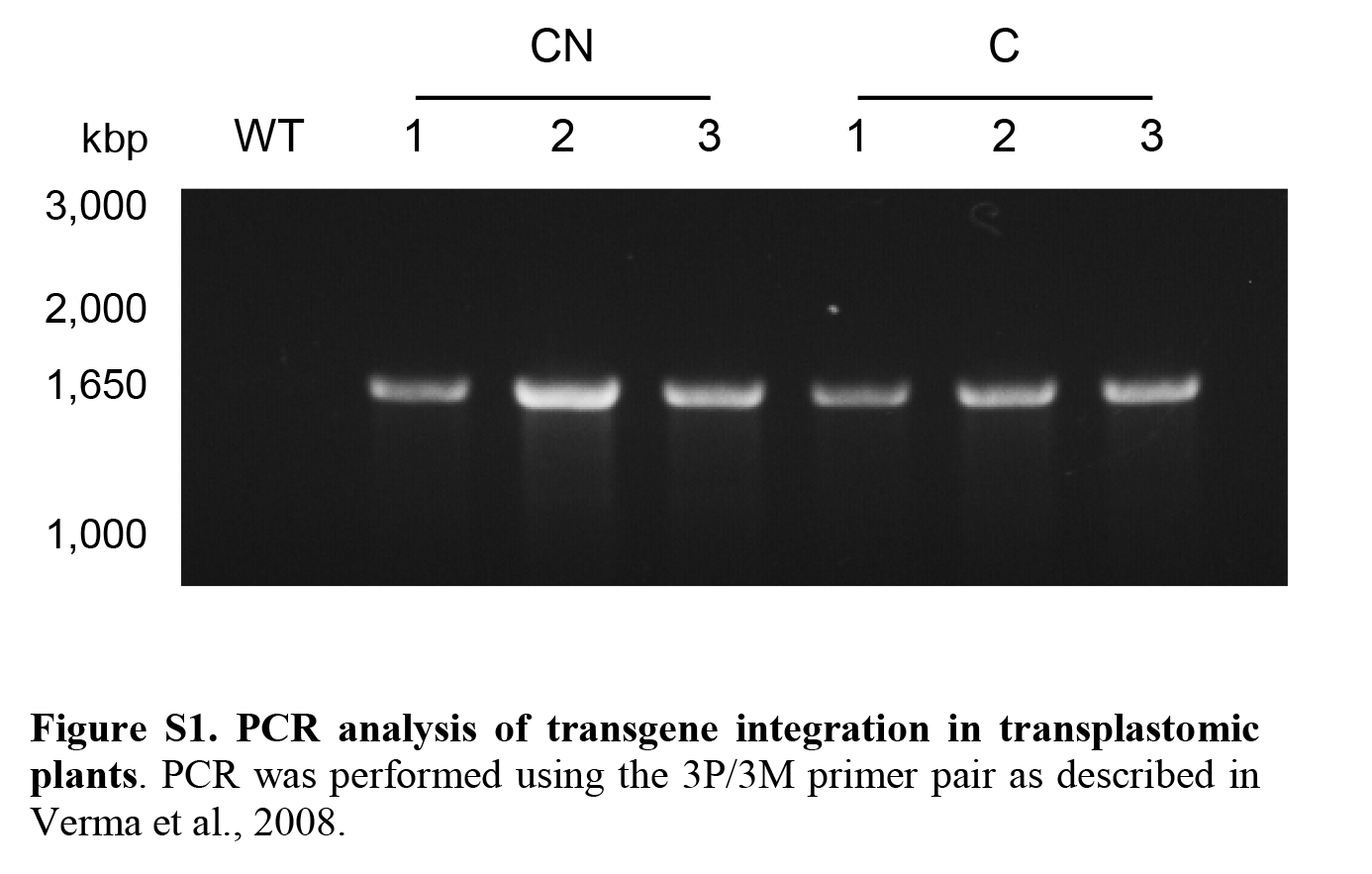

Supplement: Supplementary file 1 — Figure S1 PCR analysis of transgene integration in transplastomic plants. [file PBI-14-1862-s006.tif]

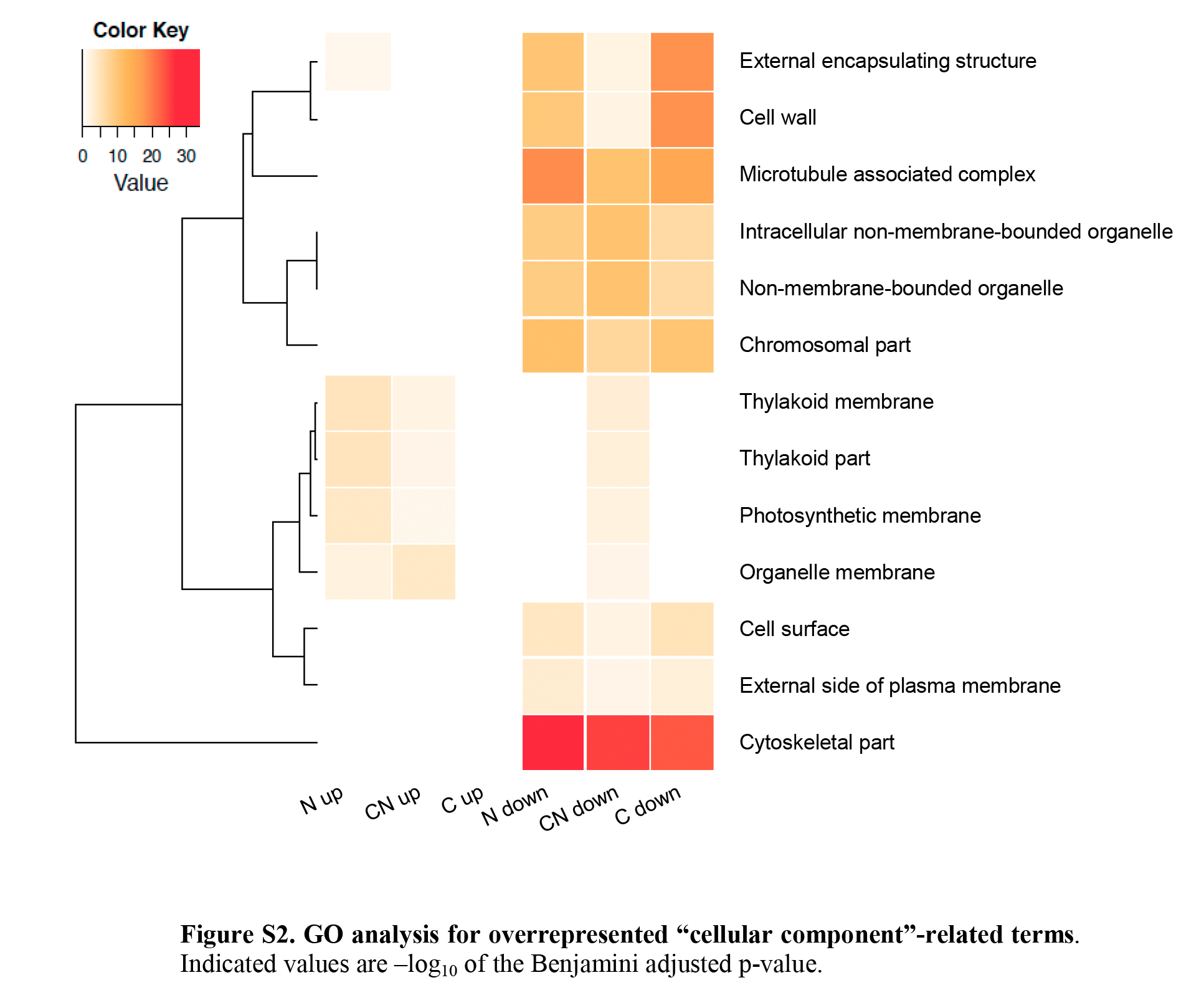

Supplement: Supplementary file 2 — Figure S2 GO analysis for ‘cellular component’‐related terms. [file PBI-14-1862-s005.tif]

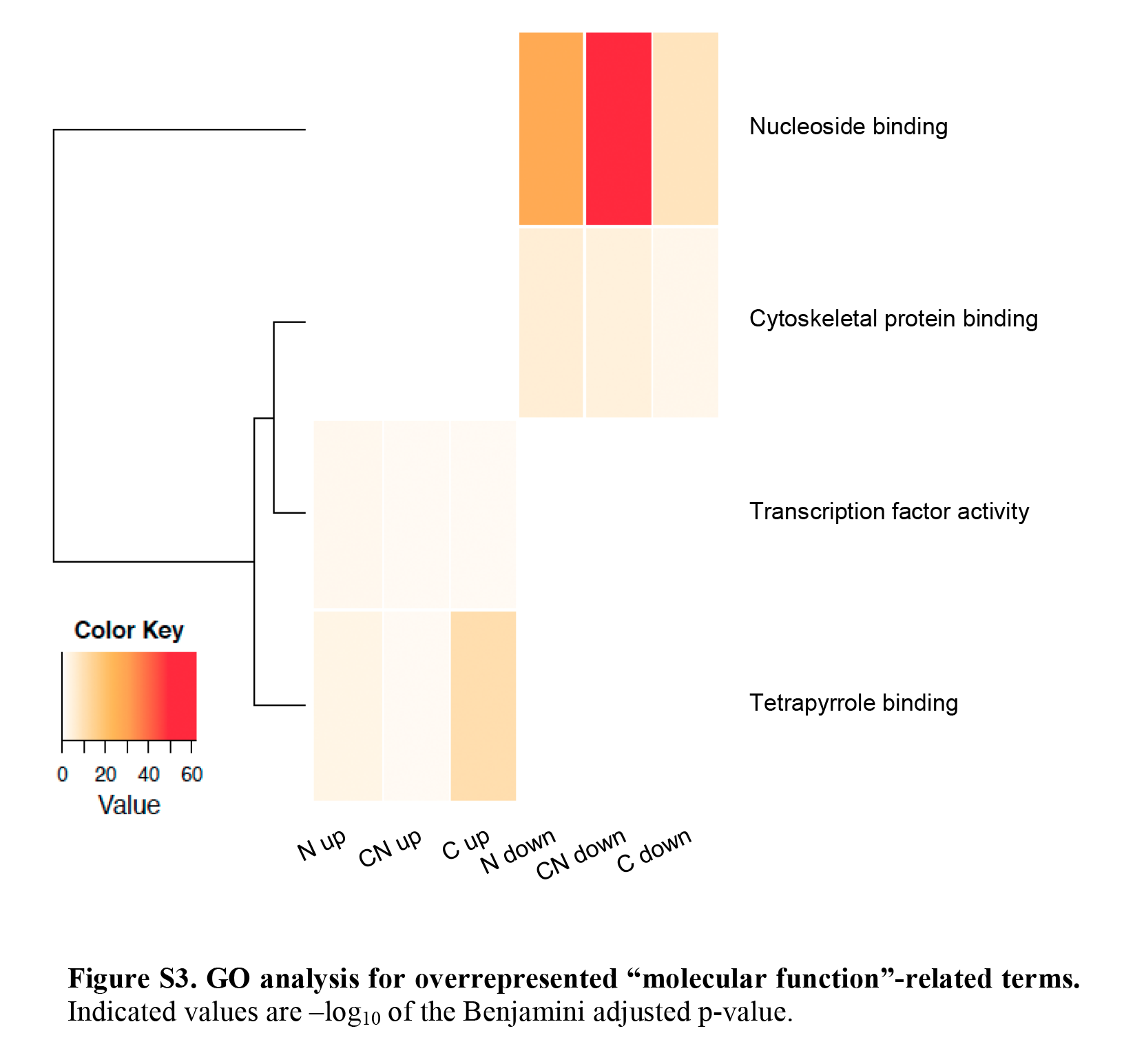

Supplement: Supplementary file 3 — Figure S3 GO analysis for ‘molecular function’‐related terms. [file PBI-14-1862-s004.tif]

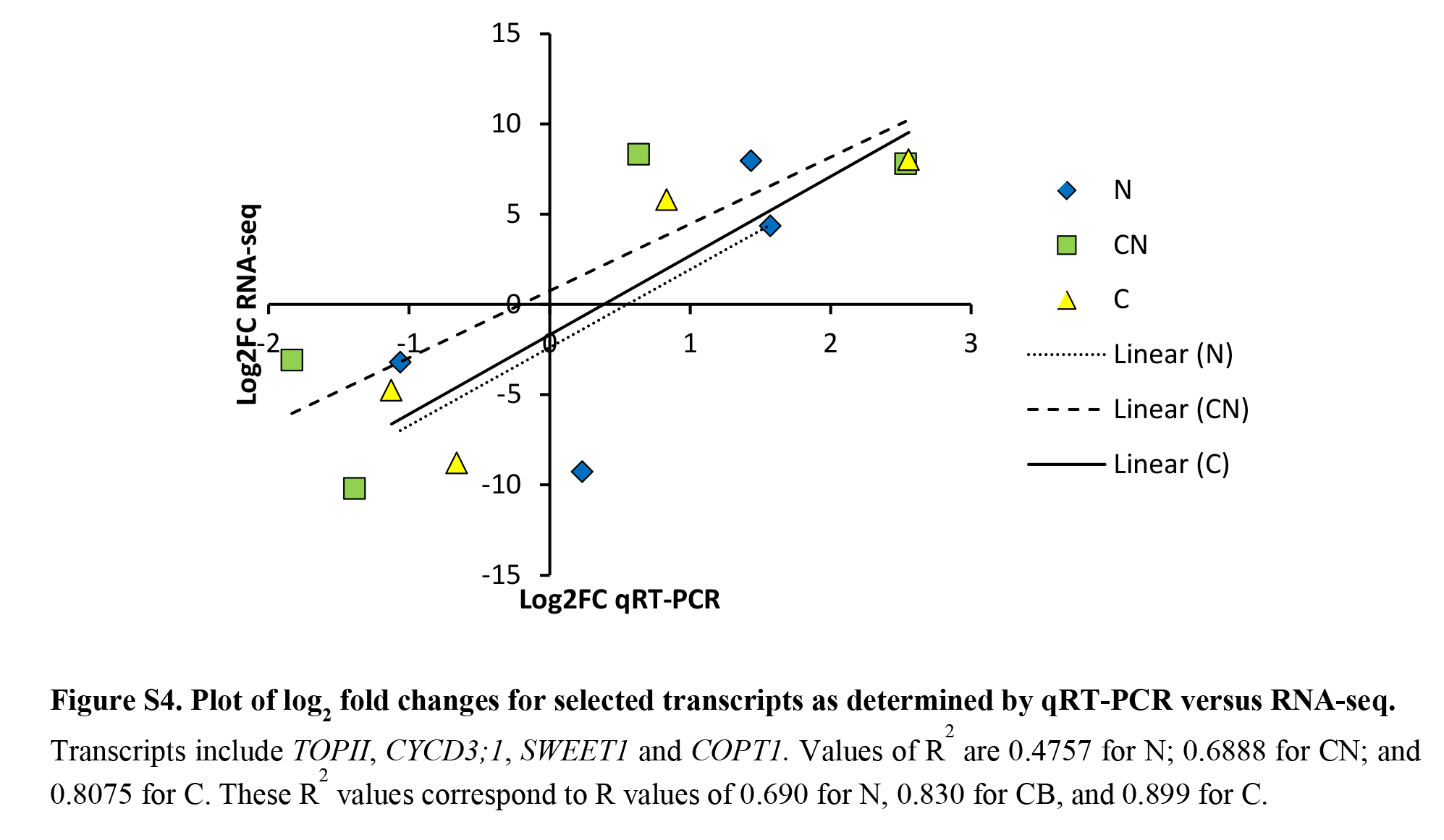

Supplement: Supplementary file 4 — Figure S4 Plot of log2 fold changes for selected transcripts as determined by qRT‐PCR versus RNA‐seq. [file PBI-14-1862-s003.tif]

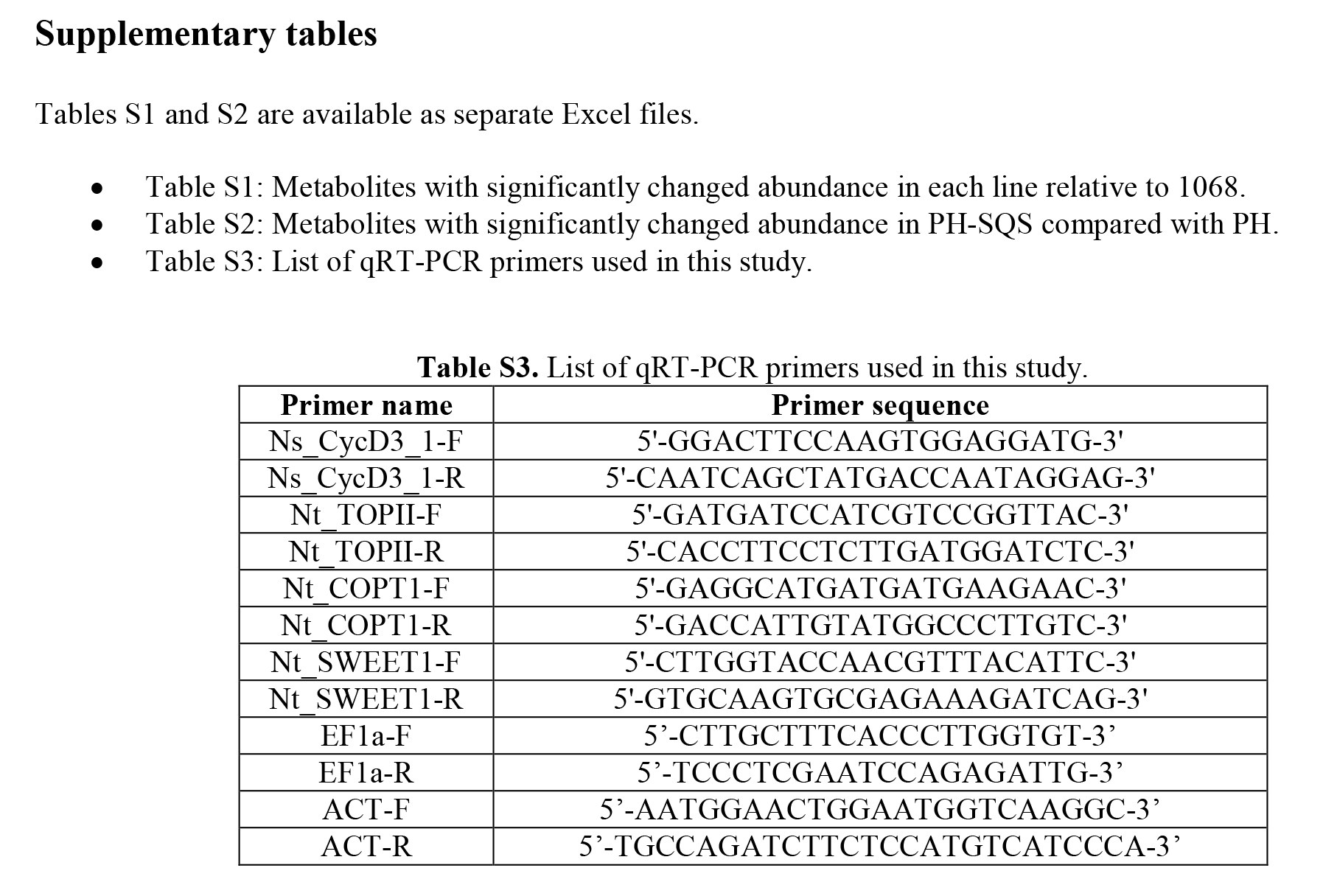

Supplement: Supplementary file 7 — Table S3 List of qRT‐PCR primers used in this study. [file PBI-14-1862-s001.tif]
